# BALCONY: an R package for MSA and functional compartments of protein variability analysis

**DOI:** 10.1186/s12859-018-2294-z

**Published:** 2018-08-14

**Authors:** Alicja Płuciennik, Michał Stolarczyk, Maria Bzówka, Agata Raczyńska, Tomasz Magdziarz, Artur Góra

**Affiliations:** 1 Tunneling Group, Biotechnology Centre, Silesian University of Technology, ul. Krzywoustego 8, 44-100 Gliwice, Poland; 2 Institute of Automatic Control, Silesian University of Technology, Akademicka 16, 44-100 Gliwice, Poland; 3 Faculty of Chemistry, Silesian University of Technology, ks. Marcina Strzody 9, 44-100 Gliwice, Poland

**Keywords:** MSA, R package, Conservation/entropy analysis, Protein evolution

## Abstract

**Background:**

Here, we present an R package for entropy/variability analysis that facilitates prompt and convenient data extraction, manipulation and visualization of protein features from multiple sequence alignments. BALCONY can work with residues dispersed across a protein sequence and map them on the corresponding alignment of homologous protein sequences. Additionally, it provides several entropy and variability scores that indicate the conservation of each residue.

**Results:**

Our package allows the user to visualize evolutionary variability by locating the positions most likely to vary and to assess mutation candidates in protein engineering.

**Conclusion:**

In comparison to other R packages BALCONY allows conservation/variability analysis in context of protein structure with linkage of the appropriate metrics with physicochemical features of user choice.

Availability: CRAN project page: https://cran.r-project.org/package=BALCONY and our website: http://www.tunnelinggroup.pl/software/ for major platforms: Linux/Unix, Windows and Mac OS X.

**Electronic supplementary material:**

The online version of this article (10.1186/s12859-018-2294-z) contains supplementary material, which is available to authorized users.

## Background

Multiple sequence alignment (MSA) and its analysis are fundamental procedures in many bioinformatics studies. MSA can contain crucial information concerning the evolution of sequences and amino acids at particular sites. Thus, variability-entropy analysis provides information on the conservation of particular residues at sites. Linking the evolution of residues to functional analysis gives insight into the strategy of enzyme adaptation [1]. Therefore, investigation of the variability of amino acids facilitates the identification of functional hot spots. Moreover, summarising the occupancy of residues at certain position in the MSA might provide important insights for protein re-engineering, when aiming to improve enzyme performance or grafting desired functionality at targeted positions. However, it is essential to evaluate not only neighbouring amino acids in primary sequence, but also dispersed ones that can be seen as neighbours in tertiary structure and can provide intrinsic functionality e.g. tunnels and gates [2].

We provide an R package that performs the unique analysis of protein evolution that summarises entropy or variability scores in an MSA. Residues responsible for protein functionality are often dispersed across the sequence but are in close proximity in the tertiary structure (we called those a functional compartment of protein structure). We have designed BALCONY (Better ALignment CONsensus analYsis) to facilitate the analysis of dispersed key amino acids, by fast and convenient data extraction, manipulation and visualization. The package has the flexibility to select amino acids and make it possible to analyse evolution of any protein structural feature constructed by residues i.e. molecular surface of protein, tunnels and molecular gates, active sites, allosteric sites, channels, switches, residues involved in protein-protein interactions interface, residues stabilizing substrates prior to reaction etc. [2–6]. BALCONY facilitates correlation of entropy/variability scores with physicochemical features of the user’s choice (e.g. collected from other bioinformatics or experimental tools). Additionally, we have included tools to provide information about the various properties of amino acids (in the so-called “grouping mode”), which coupled with statistical tools indicates selective permissibility of substitutions on investigated positions. The range of possible analyses can be extended far beyond structure/conservation data. For example, Molecular Dynamics (MD) simulations can be used to calculate mean atomic fluctuations and then it can be processed by BALCONY tools. Thus, the package can be used in thermostability optimisation, binding properties enhancement, ligands filtering analysis by channel and tunnels or in identification of hot spots for directed mutagenesis. This tool is unique in the number and kinds of analyses it implements, and also in its convenient combinability with other packages within the R environment.

The BALCONY package implements multiple entropy/variability scores that may be used to locate evolutionarily variable or preserved residues. Moreover, we are proposing a new score for the measurement of a residues conservation.

## Implementation

**Fig. 1:**
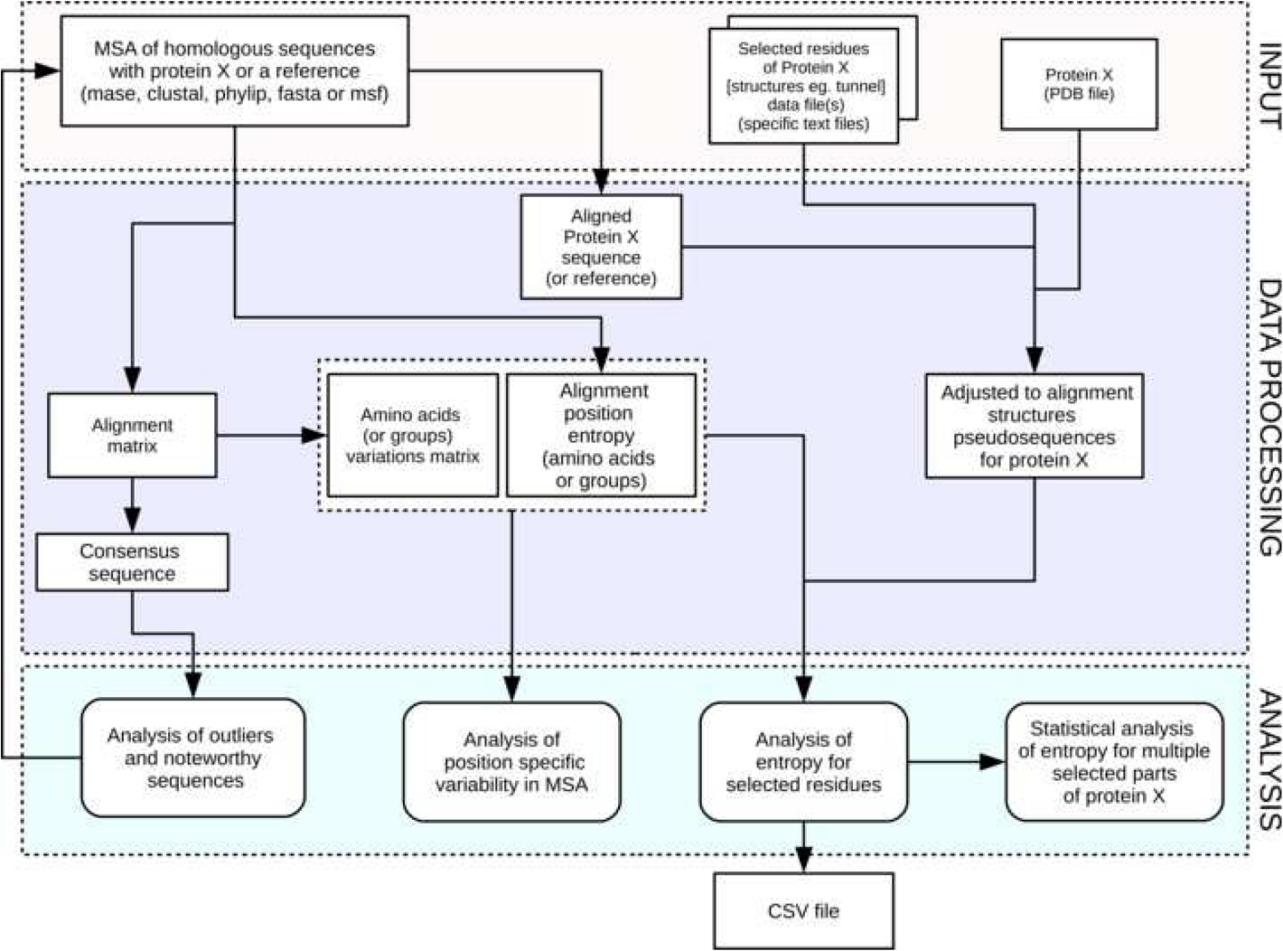
Flowchart illustrating the BALCONY package components and workflow

BALCONY is an R package available via CRAN project. Installation is straightforward and can be easily completed with standard R package installation commands. Components of the package can be grouped into three categories: data input, processing, and analysis – see Fig. 1. Results of the analysis can be saved as Coma Separated Values (CSV) file and/or can be readily plotted with dedicated functions.

BALCONY can process three different types of data: MSA of homologous sequences; specific structure/feature of protein of interest; structure of protein of interest. MSA of homologous sequences is required for all types of analyses, the other data is optional. Details of data formats used by BALCONY are provided in the BALCONY manual file.

The middle section of Fig. 1 illustrates main data processing workflows in BALCONY. For example, MSA of homologous sequences can be submitted to validation routines finalized in analysis of outliers and noteworthy sequences. It may further result in the improved MSA. Next, altered MSA data can be used in the remaining types of data processing and analysis procedures. BALCONY data processing workflows are very flexible; all functions can be easily combined, rerun, or even, if necessary and possible, run in any order.

Analysis functions allow convenient preparation of different types of plots and statistical analysis. Detailed results of entropy analysis for each alignment position can also be saved as CSV file (Additional file 1: Table S1).

In comparison to other R packages [7, 8] BALCONY facilitates summarization and conservation-variability analysis in context of protein structure. Solution presented in BALCONY package aids the comparison of functional compartments across one protein and facilitates multiple proteins statistical analysis. Also, a unique feature is the possibility to add details to investigated residues for integrative analyses.

## Results and discussion

In order to perform full evolutionary analysis coupled with structural data, the input is minimal: an MSA and a plain text file with any structural/physicochemical data provided by the user (Additional file 1: Figure S1). Both are described exhaustively in the BALCONY manual.

Initial data analysis and validation: The package allows to primarily extract, analyse and summarise information contained in the MSA. Undeniably, the quality of the alignment is essential for the downstream analyses, therefore we equipped BALCONY with features improving verification of the MSA. BALCONY aids detection of the outlying sequences. Managing outliers may be iterative process, requiring realigning sequences. Outliers that contain additional domains or large insertions and may be detected using the pairwise similarity of each sequence in the dataset to the consensus sequence. Other outliers may be detected using pairwise distances between all sequences. The user decides if outlier sequence should be removed, trimmed or left for further analysis.

The variability-conservation oriented investigation of some functional compartments of structure (residues responsible for protein functionality are often dispersed across the sequence but are neighbouring in tertiary structure) facilitates rational protein design [9, 10]. When the residues are adjacent in the sequence, obtaining this information is easy. The BALCONY package aids the entropy/variability analysis for sectors of the protein structure, which are dispersed in the primary structure. For this purpose, the special (but simple) text file containing table with numbers, names and additional numeric features of certain residues in protein structure may be used (Additional file 1: Figure S1). This functionality promotes the investigation of the evolutionary background of some functional residues in protein structure. Therefore, it may improve the process of rational protein design by easily comparing the variability of different functional compartments of a structure (like cavities, tunnels, surface, binding sites) with additional features obtained from different methods. For example, the additional numeric feature can serve information about physiochemical properties of particular amino acids, its accessibility for solvent exposure, β – factors, RMSF (Root Mean Square Fluctuation) data from MD (molecular dynamics) simulations etc. This is also suitable to compare in this way functional parts of multiple protein. Since other software for structural analysis of protein sometimes lacks the functionality for missing residues detection, BALCONY allows to correct the numbering of residues in file described above using information from the REMARK465 section in PDB file [11]. (Details in BALCONY manual).

### Estimating residue conservation - variability scores

BALCONY is the R package that calculates entropy scores for protein conservation studies. The following different metrics are available:

1. Landgraf - a metric applying the evolutionary information from substitution matrices [12]; This score allows to weigh sequences and uses Gonnet substitution matrix, which provides asymmetrical values for residue substitutions.
2. Cumulative relative entropy - this metric takes into account the evolutionary relationship between sequences measured by pairwise distances [13].
3. Real-valued Evolutionary Trace (RET) – the score which combines the evolutionary trace score and entropy measure [14]. This score cumulates information about variability in subgroups created by dividing the evolutionary tree of sequences present in MSA.
4. Shannon - a metric derived from information theory, which is simple but non intuitive for amino acids purposes [15];
5. Schneider - a normalized Shannon’s entropy by number of amino acid types [16]; This is intuitive score for measure entropy with range 0–1, but it may not play well if the number of sequences is smaller than the number of residue types.
6. Kabat - the first widely accepted symbol frequency score for the analysis of aligned protein sequences [17]; Is similar to our provided score, but it has no place for managing gaps.

We also implement a new score that differs from those presented above by treating gaps as a new letter, in a way where gaps do not alter the entropy score itself. This provides better distinction in entropy at position where gaps appear. The entropy value for residues with similar frequency of dominating amino acid will be lower if there are more residue types on this position. Our normalized metric (*E*_*score*_) is calculated with equations:

$$ {P}_i=\frac{\mathit{\max}\left({p}_i\right)}{n_i} $$

$$ {P}_i^{norm}=\frac{P_i}{\mathit{\max}(P)} $$

$$ {E}_{score}=\frac{-\mathit{\ln}\left({P}_i^{norm}\right)}{\mathit{\max}\left(-\mathit{\ln}\left({P}_i^{norm}\right)\right)} $$

where: *P*_*i*_ = amino acids frequency on *i*^th^ position where gaps are included; *n*_*i*_ = amino acids count on *i*^th^ position where gaps are excluded.

For more information about properties of scores and their comparison see [14, 18–20]. Other measures, if available in R environment, (for example one available in package ‘entropy’ [8]) can be easily incorporated into BALCONY workflow. Users can use variability/conservation/entropy data of their choice and run BALCONY functions to process it.

Analysis with BALCONY can be performed in standard or group mode. In the latter case, additional information about particular amino acid’s properties (i.e. based on BLOSUM62 substitution matrix, hydrophobicity, etc. – Additional file 1: Table S2) is included in the analysis. This facilitates the inspection of the evolution of the properties of amino acids.

Since the characteristics of residue substitution are related to function, the entropy/variability of residues may be a clue to determine its function. Different entropy metrics show variable performance to different purpose of evaluating sites in MSA. The implemented conservation metrics perform dissimilarly. Hence, the selection of entropy/variability measure should be deliberated. For example, scores which employ the background distribution of amino acids are more sensible for detecting catalytic sites, but measures counting similarity of chemical properties of residues may give false positives [19]. Other types of analyses, like identification of functional peptides, are better suited for the non-entropy metrics [21]. The BALCONY package implements different variability scores to satisfy multiple purposes of residues variability studies.

In fact, an MSA should be seen as a sample (often not perfect and reliable) of related sequences that aims at estimation of the actual composition of amino acids at any given peptide site. Notably, as a substantial basis for the downstream analyses the MSA reliability is of particular concern. In order to avoid taxonomic bias, which may strongly influence results, BALCONY allows to use sequence weighting in most of the implemented variability scores. Weighting is available in all methods using Henikoff and Henikoff position based method [22] but cumulative relative entropy. Real valued evolutionary Trace method infers weights internally using evolutionary tree generated with UPGMA (Unweighted Pair Group Method with Arithmetic Mean) method [14, 23]. Additionally, BALCONY incorporates pseudo-count method [22, 24] which allows to calculate reliable scores for MSA positions with limited number of sequences. Pseudo-counts are available for Shannon, Schneider, Kabat, and *E*_score_ methods.

### Data presentation

The package facilitates the presentation of the results as a table, which facilitates a quick and convenient investigation of the entire protein and the regions of interest. The columns of the table correspond to consecutive alignment positions. Therefore, they map the residues in the analysed protein to the position in the alignment.

**Fig. 2:**
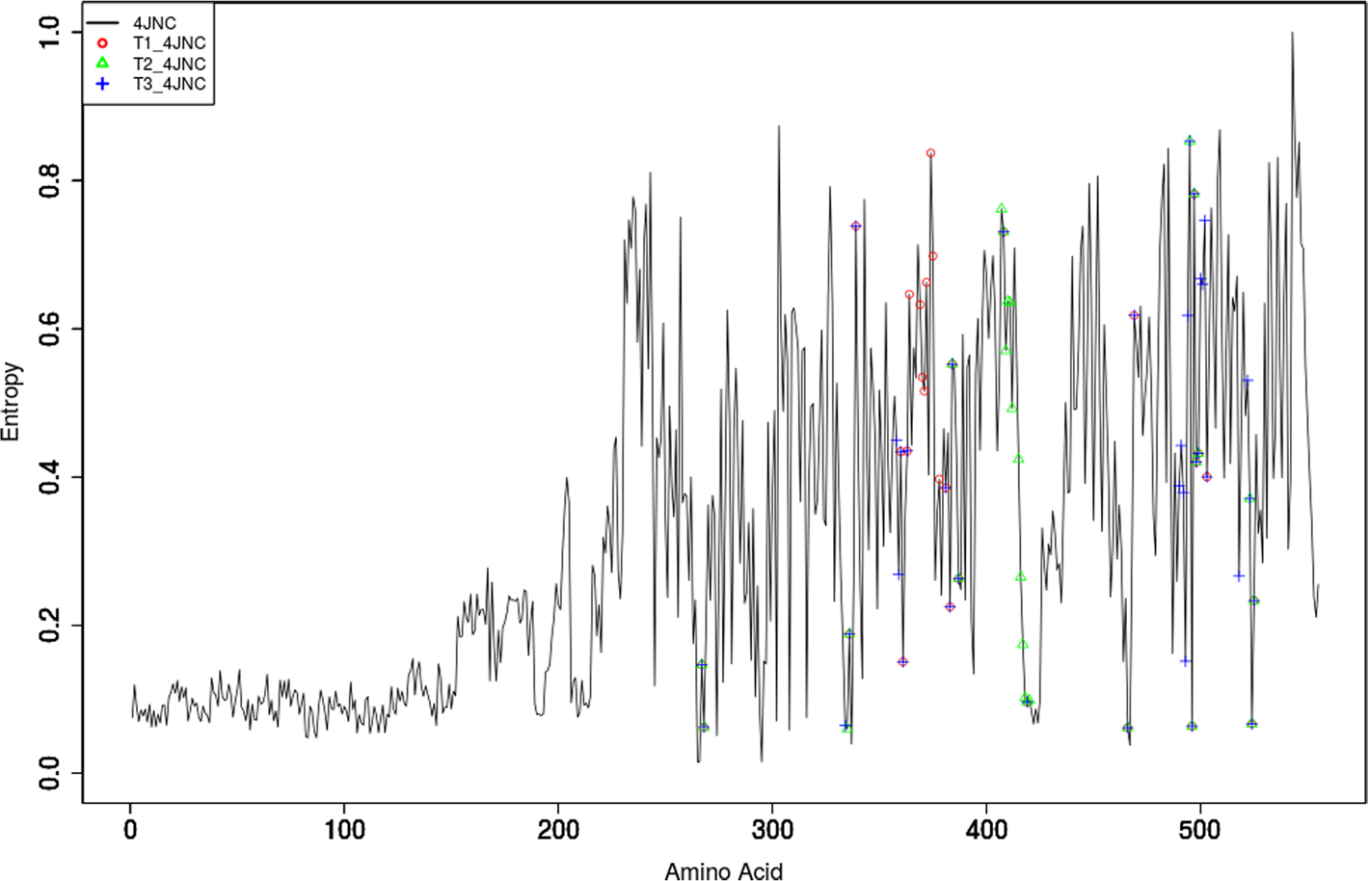
Real-valued Evolutionary Trace entropy for the whole sequence of the human soluble epoxide hydrolase, PDBid: 4JNC, UNIPROTid: P34913. Amino acids forming tunnels are marked with symbols. The part of the plot with visibly lower values of entropy at the beginning belongs to the phosphatase domain

**Fig. 3:**
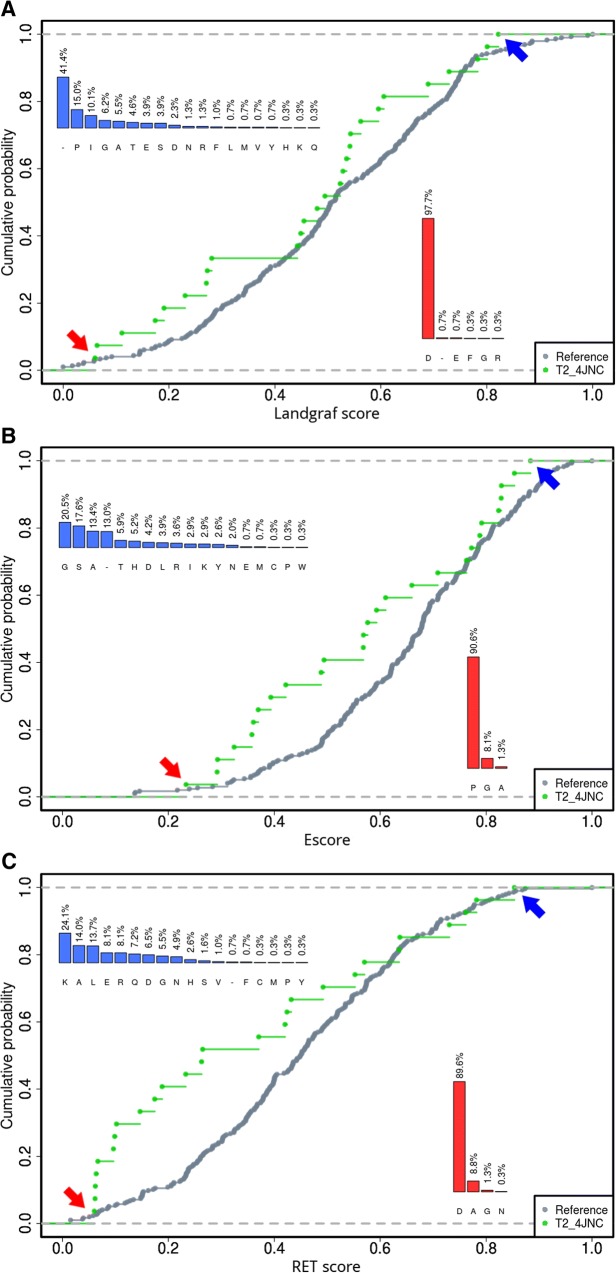
Cumulative Distribution Function plots of Landgraf (**a**), *E*_*score*_ (**b**), and Real-valued Evolutionary Trace scores (**c**). Green traces represent amino acids building tunnel T2, and grey traces represent the rest of the protein. The least variable positions in the tunnel are marked with red arrows and the most variable positions with blue arrows. The red bar plots provide information about variability of the least variable positions in the alignment, positions 1287 (**a**), 931 (**b**), 1025 (**c**); the blue bar plots about the most variable positions in the alignment, positions 1141 (**a**), 1136 (**b**), 1286 (**c**). Details in the text

The package allows for quick detection of the artefacts in the aligned sequences. Protein can have multiple functional domains, and analysis focused on one domain sometimes requires to trim MSA to remove the unnecessary ones. From the viewpoint of analysis focused on one particular functional domain, indels may be interesting but large insertions (whole domains) may obscure results. Plotting entropy/variability for alignment facilitates the detection of domains which will be visible as region of lowered (by presence of gaps) entropy. For instance, additional domains or large insertions visible as regions with distinct entropy level (Fig. 2). Results of the analysis can be plotted automatically (bar charts displaying the variability profile of MSA position (Inserts on Fig. 3), entropy profiles for entire protein for each implemented score (Additional file 1: Figure S2), which can be additionally enriched by information regarding residues that make up a region of interest (Fig. 2). The scatter plots of entropy scores facilitate the entropy metrics performance assessment (Additional file 1: Figure S3), which is imperative since there is no agreed-upon “gold standard” in quantifying evolutionary conservation at an aligned position [16]. Finally, BALCONY facilitates the comparison of evolutionary rates of particular regions using a cumulative distribution function (Fig. 3).

### Use case

To provide an example of BALCONY functionality we present a short, yet informative study. The data used to perform the analysis presented below regarding both MSA of homologous sequences to human soluble epoxide hydrolase and example of structural data are provided with the package as R dataset. We focus on the analysis of amino acids building tunnels in human soluble epoxide hydrolase (sEH). Information about tunnels and tunnel lining residues was obtained with CAVER 3.02 software [25]. Tunnels were calculated against 50 ns MD simulation, appropriate protein structure was obtained from PDB (PDB ID 4JNC) [26]. The alignment was prepared from sequences available in the UniProt database [27]. The technical details concerning analysis are available in BALCONY manual available in Additional file 2; MSA and tunnels data are included in the BALCONY package.

The alignment data in FASTA format was used in the downstream analysis. All protein isoforms in the set of aligned sequences were deleted. The threshold for the consensus calculation was set in order to select the most frequent amino acids at position but with minimum frequency over 30%, as a common rule is that sequences are homologous if they are more than 30% identical. UniProt id for 4JNC was combined with that of PDB id in order to create a mapping library. Then the consensus sequence was calculated. Amino acids classified in MSA as outliers, whose percentage of similarity between consensus and aligned sequence was small, were excluded from the analysis. In order to apply the representative group of amino acids, the consensus sequence similarity for grouped alignment was calculated. For each amino acid and grouped amino acid variation for each aligned position was calculated. The reference sequence was found from the previously created mapping library. This approach allows for convenient analysis of different protein variants without interfering with the MSA.

The entropy of each of the multiple sequence aligned position was calculated. Among all available scores in the R BALCONY package (Kabat, Shannon, Schneider, Landgraf, *E*_*score*_, Cumulative Relative Entropy, Real-valued Evolutionary Trace) were applied for comparison of their characteristics. It is due to the user which entropy score is applied for the analysis, as there is no rigorous mathematical test for judging a conservation measure. Choice of the proper conservation score may be based on the following criteria: mathematical properties, amino acid frequency, stereochemical properties, number of gaps, sequence weighting [15]. From the implemented scores Landgraf, *E*_*score*_ and Real-valued Evolutionary Trace were used as representative for study case.

Figure 2 shows plot of the Real-valued Evolutionary Trace entropy (conservation) scores of the protein with the marked amino acids which build walls of the second identified tunnel in MD simulations. The clearly visible regions with low entropy values at the beginning of the plot were identified as a the ones belonging to the phosphatase domain of the sEH family. They were removed from further analysis as it was focused on the hydrolytic domain of human sEH protein structure available in PDB [26] only. It is also clearly visible that tunnels are built by both very variable and very conserved amino acids, which were located only in the hydrolytic domain.

To investigate different rate of evolution of amino acids building tunnels with the entire protein the Kolmogorov-Smirnov non-parametric test was performed (Table 1). The null hypothesis was that the true distribution function of analysed tunnel is equal to the distribution function of the rest of the structure. *P*-value obtained from the Real-valued Evolutionary Trace is lower than the defined significance level (0.05), which leads to the conclusion that the entropy profile of the 2nd tunnel is different than the rest of the structure and this tunnel was selected for further analysis of tunnels lining residues and scores comparison with the use of Cumulative Distribution Function (CDF) - Fig. 3. Analysis of positions with high entropy value facilitates locating possible targets to introduce single-point mutations.

**Table 1:** The results of the Kolmogorov-Smirnov non-parametric test for Real-valued Evolutionary Trace scores of amino acids building tunnels in comparison with the entire protein. Details in the text.

	T1	T2	T3
RET	0.303	0.013	0.097

The results indicate that selected entropy scores provide distinct results. Different positions of both the least and most variable amino acids in the analysed tunnel were provided. Nevertheless, the results are appropriate, since the least variable amino acids occur at the particular position at around 90%. For the most variable positions there is a difference in treating gaps according to Landgraf, *E*_*score*_ and Real-valued Evolutionary Trace scores (Fig. 3a-c). Both Landgraf and *E*_*score*_ scores emphasise the possible gap occurrence, since the probability of their presence on the position is high, whereas for Real-valued Evolutionary Trace the probability of gap presence is rather low. In the case where gaps are frequent, usage of Landgraf and *E*_*score*_ are recommended. Comparison of the curves of reference suggests, that where user is interested in variable residues, the RET or Landgraf score would provide higher resolution (the differences between calculated scores are higher) and analysis of conserved one can be facilitated by *E*_*score*_.

## Conclusions

BALCONY facilitates the reading, processing and visualization of evolutionary conservation data originating from an MSA. Importantly, BALCONY can be easily integrated with other R packages that provide a coherent data flow in downstream and upstream analyses of biological sequences.

The distinctive feature of the package is the convenience in which dispersed residues in the protein sequence can be studied. Some regions of protein structure require particular investigation in terms of evolutionary variability. They might be interesting from the viewpoint of enzyme design, providing information about the evolution of residues important for protein properties (e.g. thermostability, selectivity, activity) and suggesting hotspots for amino acids substitutions. Not only the hotspots for mutagenesis are obvious, but also substitutions that enhance, degrade, or are potentially neutral to particular features.

It is worth adding that the input file facilitates linkage of calculated entropy/variability scores to other features of analysed amino acids. User has absolute freedom with decision of the source of the additional data, it can be taken from other bioinformatics tools, computational tools, but also can be taken from crystal structures information (e.g. β – factors), other experimental measurements. To provide an example of BALCONY usage, we have performed an evolutionary analysis of residues building the main tunnel of human soluble epoxide hydrolase. As the additional feature, the occurrence of particular event during molecular dynamic simulations was used. The example is available in the BALCONY manual file, together with a detailed installation guide and package description.

## Additional files

Additional file 1: BALCONY Supplementary Materials. (PDF 164 kb)

Additional file 2: BALCONY manual. This document provides a description of package installation, an example of BALCONY analysis workflow and case study. (PDF 490 kb)

## Abbreviations

CDF: Cumulative Distribution Function
CSV: Coma Separated Values
MD: Molecular Dynamics
MSA: Multiple sequence alignment
PDB: Protein Data Bank
RET: Real-valued Evolutionary Trace
RMSF: Root Mean Square Fluctuation
sEH: soluble epoxide hydrolase
UPGMA: Unweighted Pair Group Method with Arithmetic Mean
