# Supplementary material for: BALCONY: an R package for MSA and functional compartments of protein variability analysis
Source: BMC Bioinformatics. 2018 Aug 14;19:300. doi: 10.1186/s12859-018-2294-z (PMC6092823; doi:10.1186/s12859-018-2294-z)
Supplement: Supplementary file 1 — BALCONY Supplementary Materials. (PDF 164 kb) [file 12859_2018_2294_MOESM1_ESM.pdf]

BALCONY Supplementary Materials

Alicja Płuciennik, Michał Stolarczyk, Maria Bzówka, Agata Raczyńska, Tomasz Magdziarz, Artur Góra

June 5, 2018

```
#Structure  4JNC
#res    AA  property
99  ASP 39163
147 TYR 39163
148 GLN 1
288 HIP 39163
127 ILE 555
145 PHE 39162
103 MET 44
```

Figure S1: Example of a structure file. This is plane text file comprising two or three column table. The first and the second columns are numbers of residues and residues nales respectively. Thirds column can be used to provide additional numeric data describing particular structural feature, here it is propensity of being tunnel building amino acids obtained with CAVER 3.02 software.

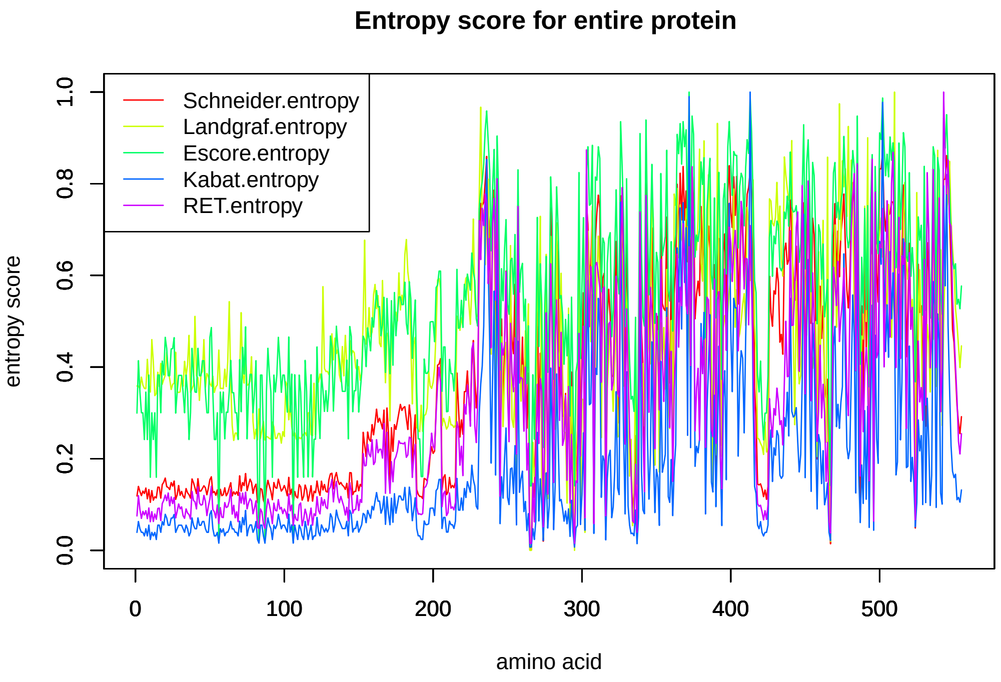

Figure S2: Plot shows the entropy (conservation) of the protein being analysed. Conservation is drastically different among regions. Additionally, it shows the differences in entropy scoring according to metrics used.

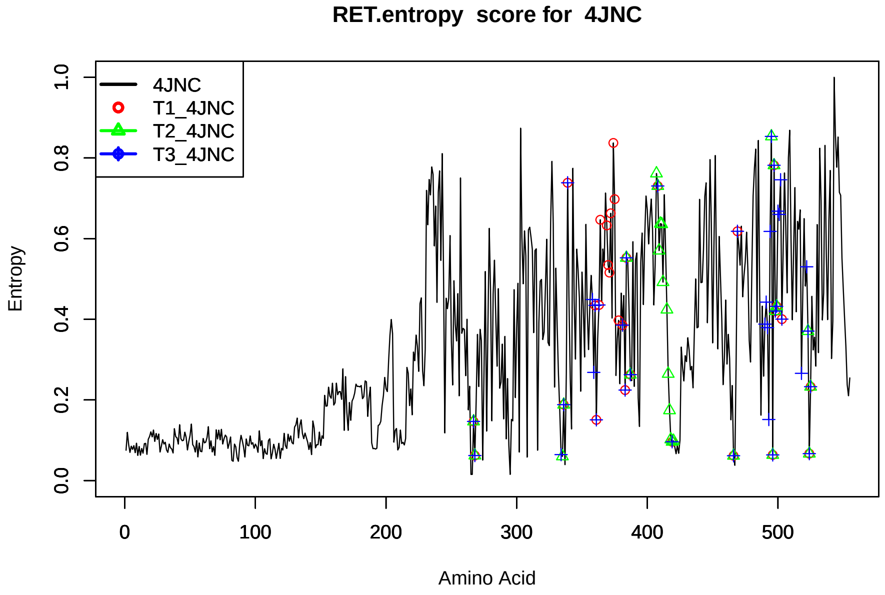

Figure S3: Plot shows the entropy (conservation) of the protein with the amino acids building structure marked on one. It facilitates the visual inspection of the chosen structural conservation in comparison with entire protein.

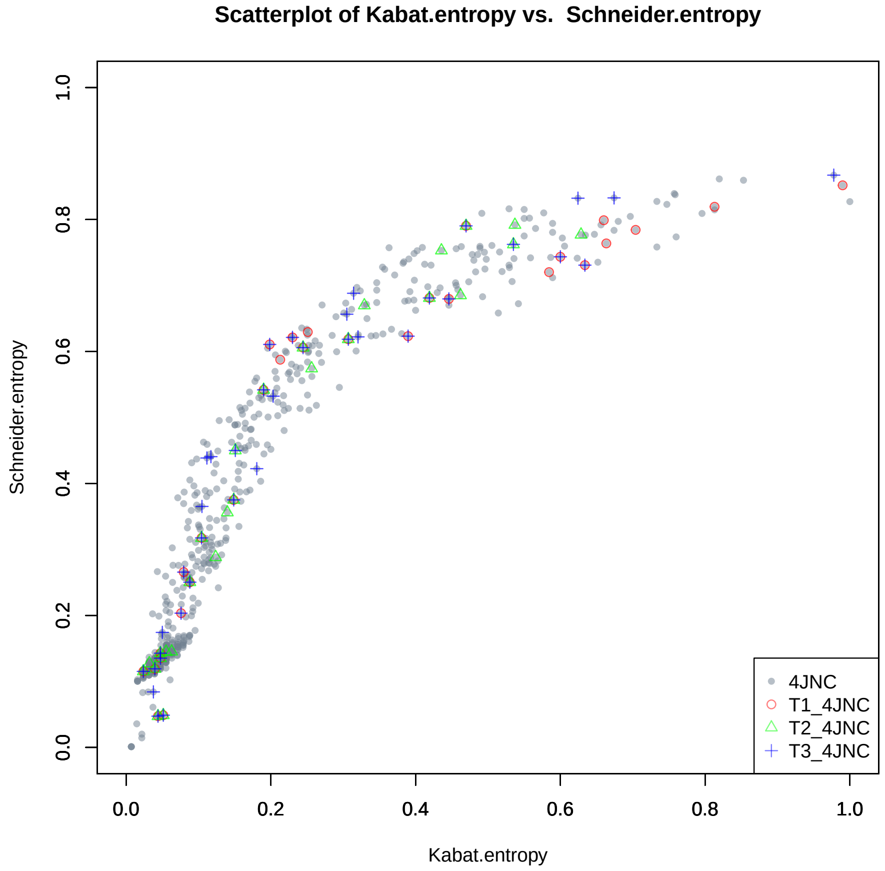

Figure S4: Scatterplot shows the relationship between a specified pair of entropy metrics.

Table S1: A sample of output CSV file.

alignment_position	1282	1283	1284	1285
AA name	I	W	G	E
Percentage	44.9511	31.9218	85.9935	33.5505
AA name	L	T	A	D
Percentage	28.6645	15.3094	9.772	25.7329
AA name	V	A	S	T
Percentage	17.9153	12.7036	2.9316	14.0065
AA name	M	V	-	A
Percentage	4.5603	9.772	0.6515	6.5147
AA name	C	H	T	S
Percentage	1.6287	8.1433	0.3257	5.5375
AA name	A	R	V	G
Percentage	1.3029	7.8176	0.3257	3.9088
AA name	-	G	n	K
Percentage	0.9772	3.2573	n	2.9316
AA name	n	n	n	n
Percentage	n	n	n	n
AA name	n	n	n	n
Percentage	n	n	n	n
sequence	v	t	a	e
structure_1	N	N	N	N
structure_2	N	N	N	N
structure_3	N	N	N	N
structure_4	N	N	N	N
Structure numbers	491	492	493	494
Schneider.entropy	0.438591455673143	0.688262034287571	0.174279963001664	0.622135015478849
Landgraf.entropy	0.49936352313306	0.900264918232074	0.200679023336388	0.578733972365186
Escore.entropy	0.525492510473891	0.751981459665551	0.356760588984607	0.756924602279168

Table S2: Summary of grouping used in BALCONY.

class	Substitution matrix	class	Size	class	Polarity and charges	class	Aromaticity
G1	A P S T	G1	A G S	G1	W S T Y N K	G1	F W H Y
G2	R Q E K	G2	V C D N T P	G1A	H R K	G2	others
G3	N D G H	G3	others	G1B	E D		
G4	I L M V			G2	others		
G5	F V Y						
G6	C						
