# Supplementary material for: BALCONY: an R package for MSA and functional compartments of protein variability analysis
Source: BMC Bioinformatics. 2018 Aug 14;19:300. doi: 10.1186/s12859-018-2294-z (PMC6092823; doi:10.1186/s12859-018-2294-z)
Supplement: Supplementary file 2 — BALCONY manual. This document provides a description of package installation, an example of BALCONY analysis workflow and case study. (PDF 490 kb) [file 12859_2018_2294_MOESM2_ESM.pdf]

BALCONY manual

Michal Stolarczyk & Alicja Pluciennik

July 19, 2018

Contents

1 Acronyms **2**

2 Introduction **2**

3 Package installation **2**

4 Basic considerations and suggestions **3**

5 BALCONY - examples of analysis **3**
5.1 MSA homology assurance 3
5.2 Residiues evolutionary variability analysis 4
5.3 Structure analysis 7
5.4 Sequence mapping 8

6 Case study **9**
6.1 Primary analysis and conservation analysis 9
6.2 Statistical analysis and visualization of the results 16

7 Grouping residues **21**

1 Acronyms

- CSV: Comma separated values
- CDF: Cumulative distribution function
- MD: Molecular dynamics
- MSA: Multiple sequence alignment
- PDB: Protein Data Bank
- sEH: Soluble epoxide hydrolase

2 Introduction

What is BALCONY?

BALCONY is an R package that facilitates the evolutionary analysis and check the variability of selected amino acids in protein structure. One of the unique functionalities of the BALCONY package concerns the analysis of individual amino acids in primary protein structure and their neighbours in tertiary structure. Residues defining active site cavities, co-factor binding sites, selectivity filters, channels or tunnels are examples of applications for this package. BALCONY is flexible and versatile enough to combine and analyse evolutionary and structural data in a simple and transparent manner.

Here we describe the optional parameters and input data that is required to perform an analysis with BALCONY. As an example to show the capacities behind the BALCONY package, we have chosen a case study of an evolutionary analysis of residues building an access pathway to the protein active site. The study of entropy of residues building tunnels performed step by step is presented in section 6.

Conservation analysis can provide insights into rational protein design. Amino acids favoured by evolution are attractive hot spots for mutations prediction. Conversely, the most variable residues may also be great candidates for enzyme property enhancements since theoretically these are not fundamental for protein stability.

3 Package installation

BALCONY package is available via CRAN project (https://cran.r-project.org/) therefore installation is straightforward. It can be installed, for example, by typing following command in the **R environment console**:

```
install.packages("BALCONY",repos = "http://cran.us.r-project.org")
```

BALCONY depends on several other packages (ie: `seqinr`, `Rpdb`, `scales`, `stats`, `dplyr`, `readr`). If you encounter problems with installation try to install required packages first. To install them please type the following line in the **R environment console**:

```
install.packages("package_name", repos = "http://cran.us.r-project.org")
```

and replace `package_name` by the name of missing package (one of: `seqinr`, `Rpdb`, `scales`, `stats`, `dplyr`, `readr`).

BALCONY also depends on `Biostrings` form Bioconductor [Pagès et al., 2016]. To install it please type the following lines in the **R environment console**:

```
source("https://bioconductor.org/biocLite.R")
biocLite("Biostrings")
```

4 Basic considerations and suggestions

The quality of MSA is essential for whole further analysis. First, internal homogeneity of MSA have to be inspected. The homogeneity means here the similarity of sequences in the analysed dataset determined by the variability of species (if the dataset is dominated by some very similar species or subspecies-like some bacterial sequences, with some other species the dataset is then very homogeneous and may lack appropriate variability information needed in further conservation analysis. Selection of sequences in the dataset is always a trade-off between high homogeneity and variability of information-rich set. Nevertheless, preparation of the dataset and MSA is usually an iterative process.

Detection of outliers in the dataset can be, for example, done by calculating identity between each sequence and the consensus sequence. This way, sequences with probable additional domains or large deletions can be easily sorted out (if necessary) since they are expected to yield larger distances. Redundant sequences can be detected with similar approach with pairwise distances calculated for all sequences.

BALCONY package can relate results to particular protein structure. If the aligned sequences are not the ones coming from structures (PDB) but from other databases (UniProt, NCBI), a mapping dictionary should be provided. It is defined as R list of vectors where the first element is a protein name in sequence alignment and the rest are PDB IDs of structures corresponding to the sequence in the alignment. It also allows to check and correct the numbering of protein residues caused by missing atoms in structure model.

Since there is no agreed-upon "gold standard" for scoring amino acid conservativity, BALCONY package offers implementation of seven different metrics. The entropies of each alignment position are used to estimate the conservation of amino acids in the protein. The scores are scaled in range 0-1. With 1 as the most variable position and 0 as the most conservative one. The conservation is simply the additive inverse of entropy value and can be estimated according to the following formula:

$$conservation = 1 - entropy$$

Please note Shannon entropy is the only score which is not normalised and can return values greater than 1.

5 BALCONY - examples of analysis

Following sections provide description of different types of calculations that can be easily performed using BALCONY package.

5.1 MSA homology assurance

5.1.1 Data required to perform the analysis

Alignment in MASE, CLUSTAL, PHYLIP, FASTA or SF format. See `read.alignment()` function from `seqinr` package [Charif and Lobry, 2007]

5.1.2 Parameters required to perform the analysis

- A threshold for consensus calculation (a number between 0 and 100). A minimal fraction of amino acids at each position across all sequences to be taken into consideration while calculating the consensus sequence. Depending on the MSA dataset heterogeneity, different consensus thresholds are applicable. The most common value for the consensus sequence is 30 percent for distantly related sequences. Higher threshold values are recommended for closely related or phylogenetically skewed taxon samples.

- Amino acids grouping method. There are a number of different grouping methods implemented in BALCONY: general, hydrophobicity, size, and aromaticity. These allow the user to group amino acids according to user interest in order to perform further analysis on specified groups of amino acids.
- Optional: a threshold for amino acid detection $\in (0, 1]$. A minimal fraction of amino acids on each alignment position in all sequences to be taken into consideration in further conservation analysis and visualization of individual alignment position variability. The default value is:

  $$p = \frac{1}{N}$$

  where:

  N - number of sequences in MSA,

  which implies inspection of all the residues present in the MSA.

5.2 Residiues evolutionary variability analysis

Most of conservativity calculation does not require any additional data besides the MSA. There are functions comparing the entropy metric's behaviour in the specific analysis. The scatter plots of entropy scores facilitate entropy metric performance assessment in the studied case, which is essential since there is no accepted objective "gold standard" in quantifying evolutionary conservation at an aligned position. Therefore, comparing the entropy score provides the proper metric determination. Therefore, the user is able to interpret the proper cut-off for a specific analysis. There are various metrics implemented in the BALCONY package (as separate functions):

- **Landgraf**: which uses Gonnet substitution matrix to estimate the dissimilarity between amino acids then incorporated to the conservation score. It is calculated according to the following formula [Landgraf et al., 1999]:

  $$Landgraf = \frac{1}{N}\sum_{i}^{N}\sum_{j>i}^{N}(w_i D(s_i(x), s_j(x)) + w_j D(s_j(x), s_i(x)))$$

  $$D(a, b) = \frac{m(a, a) - m(a, b)}{m(a, a)}$$

  where:

  N - the number of sequences in the alignment

  $s_i(x)$ - the amino acid at position x of the i-th sequence

  w_i - the weight of sequence s_i

  $D(a, b)$ - the dissimilarity of the amino acids a and b

  m - the Gonnet substitution matrix

- **Cumulative Relative Entropy**: which is calculated according to the following procedure [Hannenhalli and Russell, 2000]:
  1. Run pairwise alignments for all available sequences in the input MSA and save scores to a matrix
  2. Calculate a distance matrix based off of the alignment scores
  3. Perform hierarchical clustering on the distance matrix (UPGMA method)
  4. Get the clusters resulting from 3.

5. Divide the alignment into `sub-groups` which are the clusters
6. Run hmmbuild [Eddy, 1998] for `whole_alignment\sub-group` and `sub-group`
7. Calculate relative entropy using these two as indicated in the Reference and repeat for each `sub-group`

$$RE_i^s = \sum_{for\ all\ x} P_{i,x}^s log \frac{P_{i,x}^s}{P_{i,x}^{s_u}}$$

where:

i - position in the alignment

x - amino acid at position i

$P_{i,x}^j$ - the profle value for amino acid x at position i of the alignment

s - sub-group

s_u - union of all sub groups excluding s

8. Calculate the cumulative relative entropy

$$CRE_i = \sum_{for\ all\ s} RE_i^s$$

- **Real-valued Evolutionary Trace**: which is calculated according to formula: [Mihalek et al., 2004]:

$$RealValET = 1 + \sum_{i=1}^{N-1} \frac{1}{n} \sum_{g=1}^{n} (-\sum_{a=1}^{20} f_{ia}^g ln f_{ia}^g)$$

where:

N - number of sequences in MSA

n - number of nodes in evolutionary tree

g - number of subgroup

a - number of amino acid type

f_{ia}^g - the frequency of amino acid type a in group g

- **Shannon**: as an often-used measure of diversity Shannon's entropy can be used for the conservation analysis of amino acids in the alignment. It is calculated according to the following formula [Shannon, 1948]:

$$Shannon = -\sum_{i}^{K} \frac{n_i}{N} log_2 \frac{n_i}{N}$$

where:

K - the number of amino types present at the aligned position

n_i - the number of times the i-th amino acid appears there

N - the number of sequences in the alignment

- **Schneider**: which is a normalised Shannon's entropy. It is calculated according to the following formula [Sander and Schneider, 1991]:

$$Schneider = -\sum_{i}^{K} \frac{n_i}{N} \ln \frac{n_i}{N} * \frac{1}{\ln K}$$

  where:

  K - the number of amino types in the alignment (21)

  n_i - the number of times the i-th amino acid appears there

  N - the number of sequences in the alignment

- **Kabat**: the first widely accepted frequency based measure of conservation, introduced in 1970. It accounts for the relative frequency of the symbol and is scaled by the number of sequences in considered alignment. It is calculated according to the following formula [Wu and Kabat, 1970]:

$$Kabat = \frac{k}{n} * N$$

  where:

  k - the number of amino types present at the aligned position

  n - the number of times the most commonly occurring amino acid appears there

  N - the number of sequences in the alignment

- $\boldsymbol{E_{score}}$: which treats gaps in the alignment as a new letter and in consequence yielded entropy scores are not altered by gaps. Proposed metric is calculated with following formulas:

$$P_i = \frac{max(p_i)}{n_i}$$

$$P_i^{norm} = \frac{P_i}{max(P)}$$

$$E_{score} = \frac{-\ln(P_i^{norm})}{max(-\ln(P^{norm}))}$$

  where:

  p_i - amino acids frequency on i-th position where gaps are included

  n_i - amino acids count on i-th position where gaps are excluded

5.2.1 Improvement of evolutionary analysis

To take into accont imperfections of MSA to obtain better estimation of evolutionary variability of residues some improvements might be applied.

- **weights**: weighting for each sequence is a comfy way to enhance signals from underrepresented sequences types in prepared MSA. Such weights might be calculated in example from distances between sequences (based on pairwise similiarities of sequences) or similarity of each sequence to consensus sequence. BALCONY implements Henikoff and Henikoff position based sequence weighting method [Henikoff and Henikoff, 1994].

- **pseudocounts**: are used to estimate frequencies of amino acids based on non-observed in particular alignment sequences. This technique is ecpecially useful when the number of sequence in MSA is small. BALCONY package implements a method to calculate pseudocounts based given alignment and substitution matrix, which is available as a parameter in implemented scores which are presented above. Default, the residue counts are taken from MSA frequencies [Henikoff and Henikoff, 1996], [Claverie, 1994].

5.2.2 Data required to perform the analysis

- Optional: a substitution matrix. This matrix is used to calculate Landgraf conservation metric. As suggested in [Landgraf et al., 1999] we use the Gonnet substitution matrix, which is attached as a dataset in the R package. If a different substitution matrix is not specified, the Gonnet matrix is used as the default.

5.2.3 Parameters required to perform the analysis

- Alignment positions to analyse. Note that the numbers of amino acids among sequences in a multiple sequence alignment are hardly ever the same. We suggest performing the analysis to the point where a results table (saved as CSV file) is generated (see Case study) that contains mapped numbers of amino acids in the sequence of interest and in the multiple sequence alignment as shown in the table 1

5.3 Structure analysis

The conservation analysis of some regions of a protein structure facilitate rational protein design. When the residues are in sequence, obtaining this information is easy. The BALCONY package aids the analysis of sectors of the protein structure, which are dispersed in the primary structure.

5.3.1 Data required to perform the analysis

To work with residues dispersed in the protein sequence a proper input file have to be prepared. The listing below shows example of such file. It is a regular text file with two or three columns comprising following data:

1. residue number - according to the reference protein sequence (the best case), BALCONY allows to correct numbering of amino acids for MSA-based study;
2. residue name - either the three letter code or the one letter code of residue, this provides for an easy comparison of the protein sector residues within the MSA;
3. Optional: the arbitrary property of a given residue provided as a number - this might be any information that allows for the specification or selection of the appropriate subgroup of the chosen protein sector. An example of such a property may be occurrences of the residues as amino acids building protein tunnel in molecular dynamics simulation(MD). Ones that were indicated only in e.g. 1% of all frames of the MD can be ignored if needed. Other properties include heavy atoms count, or even a binary number, e.g. capability of creating a hydrogen bond.

Depending on the source of sectors in protein structure, the numbering of residues might differ (possibly caused by extra chains in sequences, missing residues within protein structure or insertion codes). Consequently, a very important stage of data preparation is the numbering of structural features with reference to the MSA sequence identity, e.g. if there are any missing residues specified in the corresponding PDB file. The methods of correcting such incompatibilities are described in section 6.2.

For example, if we take a piece of a protein tunnel (more specifically - residues forming the tunnel) from the 4JNC structure (human soluble epoxide hydrolase) [Thalji et al., 2013] detected with CAVER [Chovancova et al., 2012] based on a 50ns long molecular dynamics simulation. The additional property in this case is

the number describing how many times residue was identified as one forming the tunnel during the whole simulation. The property value facilitates the removal of the amino acids of lower importance from the further analysis.

The structure files should be formatted as presented below (with optional hash marks (#) for comment lines):

```
#Structure  4JNC
#res    AA  property
99  ASP 39163
147 TYR 39163
148 GLN 1
288 HIP 39163
127 ILE 555
145 PHE 39162
103 MET 44
```

Please note that with BALCONY, one can analyse one or more structures (protein sectors), depending on how many are read with the `read_structure()` function. More details regarding reading structure files are presented in section 6.

5.4 Sequence mapping

If the aligned sequences are not the ones coming from crystal structures (PDB) but from additional databases (UniProt, NCBI), a mapping dictionary should be provided.

5.4.1 Data required to perform analysis

- MSA,
- Mapping dictionary (a list of vectors where the first element is a protein name in sequence alignment and the rest are PDB IDs of structures corresponding to the sequence in the alignment).

```
dictionary = list(
  c("P34913","4JNC"),
  c("P34914","1EK2","1CR6","1EK1","1CQZ")
  )
```

As the example of such mapping dictionary we present P34913 and P34914 which are UniProt IDs of human and murine soluble epoxide hydrolases (sEH) and the corresponding PDB structures.

5.4.2 Parameters required to perform the analysis

- The name of the molecule studied in the MSA. The analysis will be directed towards this protein. If the aligned sequences are not the ones coming from structures but from additional databases, the name is the first element of the vector in the mapping dictionary. Otherwise, use the name of the sequence from the alignment.
- A path to PDB file, required in cases of missing structural elements of the analysed protein (e.g. a missing loop).
- A sequence shift. In case of a missing amino acid(s) in the crystal structure sequence (PDB) compared with one used in sequences alignment, the shift must be provided. We suggest checking for any shift by performing the pairwise alignment of the sequence of the structure and the corresponding sequence from the MSA. Alternatively, the shift can be provided automatically, if information about missing residues can be found in remark 465 in REMARK section of PDB file [Berman et al., 2003, PDB]. **Please note** that if the PDB structure sequence is incomplete, and amino acids numbers are taken from other software, some rearrangements may occur.

6 Case study

In the present case study, we focus on the human sEH tunnels, determined in the 4JNC structure and the alignment of its close homologous sequences available in the UniProt database [noa, 2017]. The data used to perform the analysis presented below regarding both MSA and structure are provided with the package as an R dataset.

6.1 Primary analysis and conservation analysis

Downloading, installing, loading BALCONY and setting the working directory

```
install.packages("BALCONY", dependencies = T, repos = "http://cran.us.r-project.org")
require(BALCONY)
setwd("path/to/working/directory")
```

Reading the alignment data in FASTA format to be used in the downstream analysis.

```
fpath = system.file("extdata", "aln2_312_pro.fasta", package = "BALCONY")
require(seqinr)
file = seqinr::read.alignment(
  file = fpath,
  format = "fasta",
  forceToLower = F
  )
```

Deleting all protein isoforms in the set of aligned sequences.

```
file = delete_isoforms(file)
```

```
## Warning in delete_isoforms(file): 4 isoforms were deleted
```

Setting PDB name. Here, we present the analysis for tunnels identified in the 4JNC structure [Thalji et al., 2013] with CAVER software [Chovancova et al., 2012].

```
pdb_name = "4JNC"
```

Setting the threshold for the consensus calculation. This way only the positions where minimum relative frequency for a character is over 30% will be returned as the consensus character. Otherwise, ”*” is returned for this position instead.

```
threshold_consensus = 30
```

Setting the grouping method for the calculation of the consensus to the peptide sequence similarity. The grouping key can be found in the table presented in section 7.

```
grouping_method = "substitution_matrix"
```

Library mapping Uniprot names to PDB structures IDs. A vector of strings where the first element is a Uniprot ID and others are available PDB structures IDs.

```
lib = list(
  c("P34913","4JNC"),
  c("P34914","1EK2","1CR6","1EK1","1CQZ")
  )
```

Calculating the consensus sequence. Make sure that the BALCONY package `consensus()` function is not covered by the seqinr package `consensus()` function [Charif and Lobry, 2007]. A good practice is to indicate the origin of this function explicitly: `BALCONY::consensus()`

```
consensus_seq = BALCONY::consensus(alignment = file, threshold = threshold_consensus)
```

Calculating the grouped consensus sequence. For this, you need to link the `consensus()` function with the grouped aligned sequences matrix.

```
grouped_consensus = BALCONY::consensus(
  alignment = align_seq_mtx2grs(
    aligned_sequences_matrix = alignment2matrix(file),
    grouping_method = grouping_method
    ),
  threshold = threshold_consensus
  )
```

Calculating the consensus sequences similarity (instead of amino acids, their group representatives are taken into consideration. Groups are established according to amino acid properties of user's choice (see table 1)

```
grouped_alignment = align_seq_mtx2grs(aligned_sequences_matrix = alignment2matrix(file),
                                      grouping_method = grouping_method)

consensus_sequences_similarity = cons2seqs_sim(grouped_alignment = grouped_alignment,
                                               grouped_consensus_seq = grouped_consensus)
```

Calculating the consensus sequence identity.

```
consensus_sequences_identity = cons2seqs_ident(alignment = file,
                                               consensus_seq = consensus_seq)
```

The following line finds the most similar and the least similar sequences to the consensus (detects outliers, which can be excluded from the further analysis).

```
noteworthy_sequences = noteworthy_seqs(percentage = consensus_sequences_identity,
                                       alignment = file)
```

Calculating the amino acids and amino acid group variations for each alignment (protein) position. This way you can visually estimate the alignment positional variability.

```
var_aa = calculate_AA_variation(alignment = file)
var_aa_grouped = calculate_AA_variation(
  alignment = file,
  grouped = T,
  grouping_method = "substitution_matrix"
  )
variations_matrix = var_aa$matrix
variations_matrix_grouped = var_aa_grouped$matrix
```

Finding the reference sequence.

```
uniprot = find_seqid(sequence_id = pdb_name, library = lib)
my_seq = find_seq(sequence_id = uniprot, alignment = file)
```

Calculating the weights of the sequences based on Henikoff and Henikoff position based method [Henikoff and Henikoff, 1994]. Weights are further used in entropy scores calculations to mitigate bias of redundant sequences.

```
sequences_weights = get_pos_based_seq_weights(file)
```

Calculating the entropy of each of the multiple sequence alignment positions: Kabat, Shannon, Schneider, Landgraf, E_{score}, Cumulative relativity entropy, Real-valued Evolutionary Trace scores. Also, all metrics except Cumulative relativity entropy and Real-valued Evolutionary Trace accept weights or pseudo-counts

parameters. Real-valued Evolutionary Trace infers weights internally using evolutionary tree. For more detailed information regarding the implemented entropy metrics see section [Conservation-entropy analysis] or the documentation to the functions used below (provided with the R package).

```
kabat_cons = kabat_conservativity(alignment = file)
shannon_cons = shannon_conservativity(alignment = file)
schneider_cons = schneider_conservativity(alignment = file)
Escore_cons = Escore_conservativity(alignment = file)
```

Please note that Landgraf, Cumulative realitivity entropy and Real-valued Evolutionary Trace metrics calculations are time consuming.

```
# landgraf_cons = landgraf_conservativity(alignment = file,
#                                          weights = sequences_weights)
schneider_cons = schneider_conservativity(alignment = file,
                                          weights = sequences_weights)
Escore_cons = Escore_conservativity(alignment = file,
                                    weights = sequences_weights)
kabat_cons = kabat_conservativity(alignment = file,
                                  weights = sequences_weights)
# CRE_cons = CRE_conservativity(alignment = file,
#                               hmmbuild_path = "/absolute/path/to/the/hmmbuild/binary")
#                               hmmbuild_path (optional if running under UNIX)
# RET_cons = RealValET_conservativity(alignment = file)
```

Writing the final output: amino acid variations, structure data and conservation scores combined in a handy CSV file. The table can be browsed both as a matrix in R environment or as a CSV file that is automatically saved in the current working directory. The table will be formatted as shown in the table below.

```
entropy_data = list(
  Kabat.entropy = kabat_cons,
  # Shannon.entropy = shannon_cons,
  Schneider.entropy = schneider_cons,
  Escore.entropy = Escore_cons
  # Landgraf.entropy = landgraf_cons,
  # CRE.entropy = CRE_cons,
  # RET.entropy = RET_cons
  )
```

```
final_CSV = create_final_CSV(
  filename = "BALCONY_OUTPUT",
  variations_matrix = var_aa,
  structure = structure,
  sequence_id = uniprot,
  alignment = file,
  score_list = entropy_data
  )
```

Table 1: A sample of output CSV file.

alignment_position	1282	1283	1284	1285
AA name	I	W	G	E
Percentage	44.9511	31.9218	85.9935	33.5505
AA name	L	T	A	D
Percentage	28.6645	15.3094	9.772	25.7329
AA name	V	A	S	T
Percentage	17.9153	12.7036	2.9316	14.0065
AA name	M	V	-	A
Percentage	4.5603	9.772	0.6515	6.5147
AA name	C	H	T	S
Percentage	1.6287	8.1433	0.3257	5.5375
AA name	A	R	V	G
Percentage	1.3029	7.8176	0.3257	3.9088
AA name	-	G	n	K
Percentage	0.9772	3.2573	n	2.9316
AA name	n	n	n	n
Percentage	n	n	n	n
AA name	n	n	n	n
Percentage	n	n	n	n
sequence	v	t	a	e
structure_1	N	N	N	N
structure_2	N	N	N	N
structure_3	N	N	N	N
structure_4	N	N	N	N
Structure numbers	491	492	493	494
Kabat.entropy	0.111594202898551	0.314285714285714	0.05	0.320388349514563
Schneider.entropy	0.438591455673143	0.688262034287571	0.174279963001664	0.622135015478849
Escore.entropy	0.525492510473891	0.751981459665551	0.356760588984607	0.756924602279168

6.1.1 Plot and inspect the amino acids variability on selected positions, e.g. 750, 1000, 1235

Position 750

```
barplotshow(position = 750, AA_variation = var_aa)
```

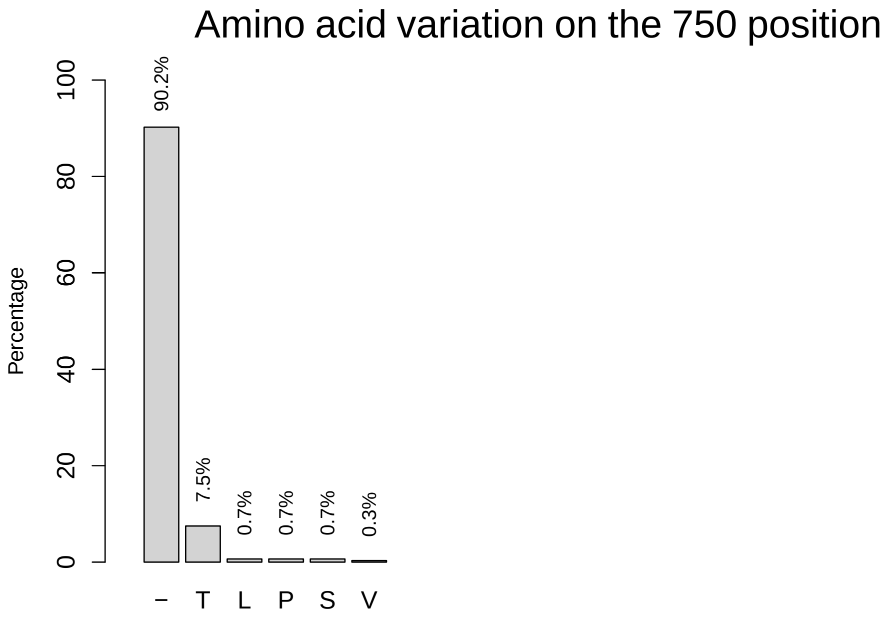

Figure 1: A bar chart presenting the percentage of each amino acid detected on the 750th position of multiple sequence alignment.

Table 2: Entropy scores for 750th position.

	Structure numbers	Kabat.entropy	Schneider.entropy	Escore.entropy
Entropy scores values	206	0.0476534296028881	0.132683635730485	0.347000727556043

Interpretation of results: The 750th column of the studied MSA contains 90% gaps. Besides, there are five other amino acids. Hence few of sequences in the MSA contain additional residues (presumably due to insertions).

Position 1000

```
barplotshow(position = 1000, AA_variation = var_aa)
```

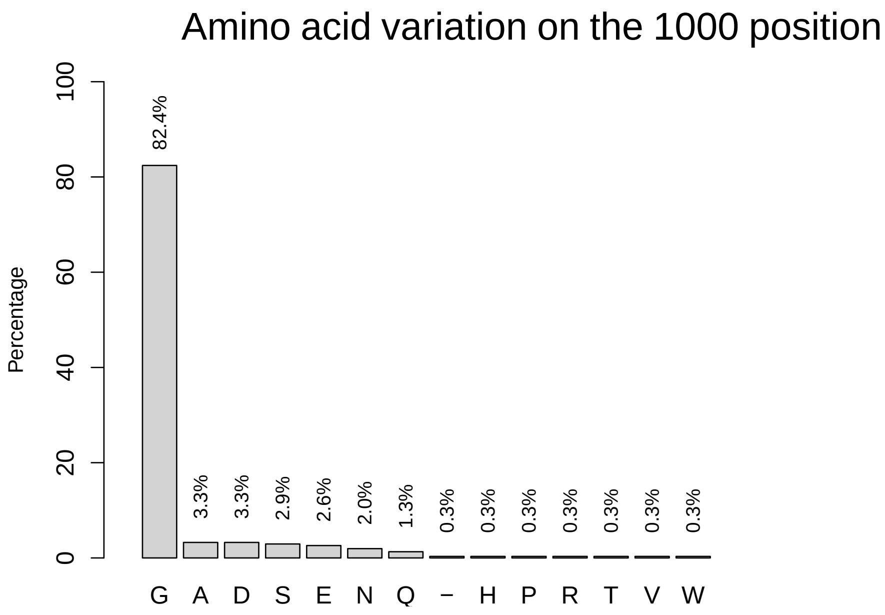

Figure 2: A bar chart presenting the percentage of each amino acid detected on the 1000th position of multiple sequence alignment.

Table 3: Entropy scores for 1000th position.

	Structure numbers	Kabat.entropy	Schneider.entropy	Escore.entropy
Entropy scores values	325	0.121739130434783	0.277566937139253	0.559411454570633

Interpretation of results: The 1000th column of MSA consists of thirteen residues and a gap, but one of them is predominant. The residue of highest frequency is Gly. Since around 18% of other residues differ in terms of size and chemical properties the Gly is the most favourable in evolution process. Nonetheless, other residues are sometimes allowed.

Position 1235

```r
barplotshow(position = 1235, AA_variation = var_aa)
```

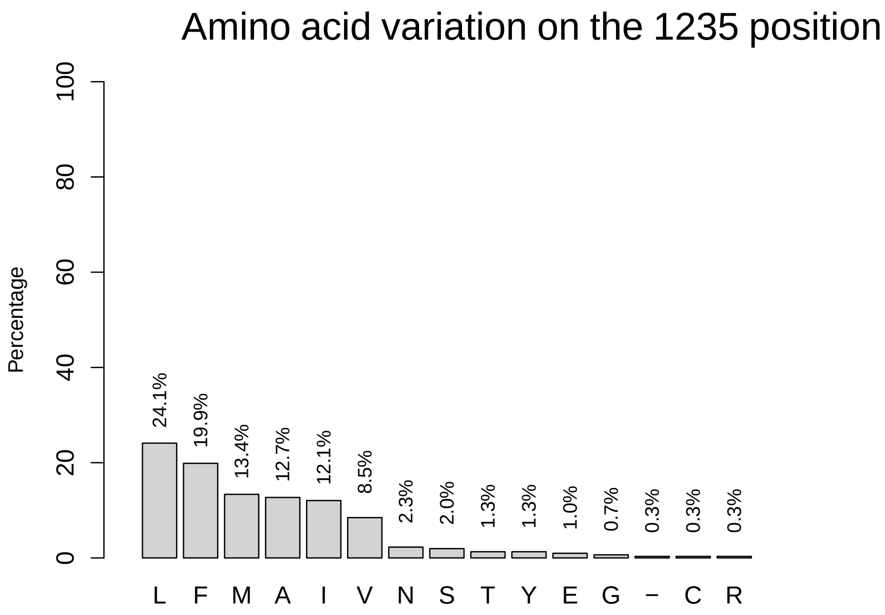

Figure 3: A bar chart presenting the percentage of each amino acid detected on the 1235th position of multiple sequence alignment.

Table 4: Entropy scores for 1235th position.

	Structure numbers	Kabat.entropy	Schneider.entropy	Escore.entropy
Entropy scores values	469	0.445945945945946	0.679687050866761	0.824063762868572

Interpretation of results: at the 1235th position of the MSA there are fifteen distinct amino acids. Consequently, this position is highly diverse. Even so, some residues tend to be more conserved than others. The variability of residues is seemingly among the hydrophobic ones.

6.2 Statistical analysis and visualization of the results

Create the structure object of the provided structural data and (optional) correct it according to the remarks comprised in REMARK465 section of the PDB file. Once you have installed the BALCONY package you are provided with the exemplary structure data that comes with it. To get the path to the data use the following line of code:

```
system.file("extdata", package = "BALCONY")
```

The output of the function used above can be used in the line below to find the exemplary structure data:

```
path = system.file("extdata", package = "BALCONY")
setwd(path)
myFiles = list.files(pattern = "*.structure")
structure_list = read_structure(file_names = myFiles)
structure = create_structure_seq(
  structure_list = structure_list,
  sequence_id = uniprot,
  alignment = file,
  pdb_path = "4jnc.pdb",
  chain_identifier = "B",
  shift = 236
  )
```

The setted shift value is a results of the way the structure was deposed in PDB. The first 236 AA were not included into structure, and are not included into section *REMARK 465*. The best way to avoid incorrect numbering is to align reference sequence from MSA with sequence in FASTA file available for PDB structure.

```
setwd("path/to/working/directory")
```

In order to enhance the structural data quality, one can exclude the rarest structural amino acids.

```
structure = excl_low_prob_strcts(structure = structure, threshold = 0.4)
```

Extract the positions of the whole protein and analysed structure (e.g. functionally related amino acids dispersed across the sequence) in the MSA based on sequence corresponding to the crystal structure.

```
indices = get_structures_idx(structure = structure)
tunnel_index = indices$structureIndices
protein_index = indices$proteinIndices
```

Get the entropy values for each analysed structure (here: a tunnel) expressed in each entropy metric

```
structure_entropy = get_structures_entropy(structure_index = tunnel_index,
                                           score_list = entropy_data)
```

Get the entropy values analysed protein expressed in each entropy metric

```
prot_cons = get_prot_entropy(protein_index = protein_index, score_list = entropy_data)
```

Visual the entropy for the whole protein

```
plot_entropy(
  protein_conservation = prot_cons,
  impose = T,
  legend_pos = "topleft"
  )
```

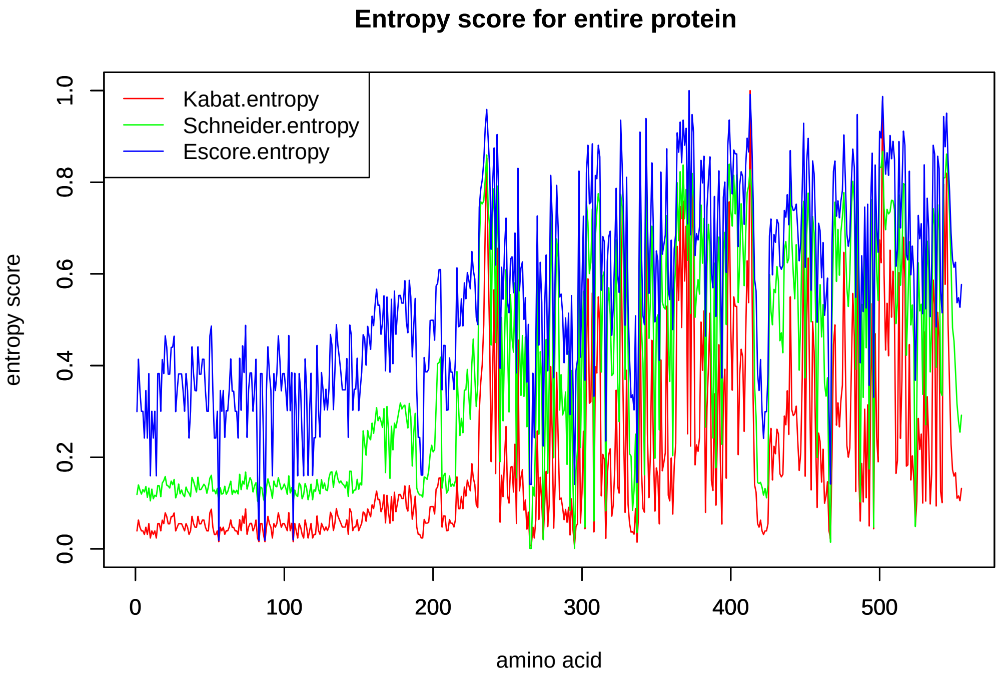

Figure 4: Plot shows the entropy (conservation) of the protein being analysed. Conservation is drastically different among regions. Additionally, it shows the differences in entropy scoring according to metrics used.

Interpretation of results: by means of the visualization of the entire protein's conservation, is was possible to detect the outlying part of the protein between amino acids residues 0 and 233, that is a phosphatase domain of the sEH. Since this domain does not contain any amino acids structures of interest, it will be excluded from the statistical analysis presented below.

Aggregate the entropy and index data into object for visualization purposes

```
profiles_for_structure = prepare_structure_profile(structure = structure,
                                                   structure_entropy = structure_entropy)
```

Visualise the structure(s) entropy on the protein background. Such a visualization facilitates the preliminary estimation of the conservation of the structure(s) of interest

```
plot_structure_on_protein(
  protein_entropy = prot_cons,
  structure_profiles = profiles_for_structure,
  pdb_name = pdb_name,
  legend_pos = "topleft"
  )
```

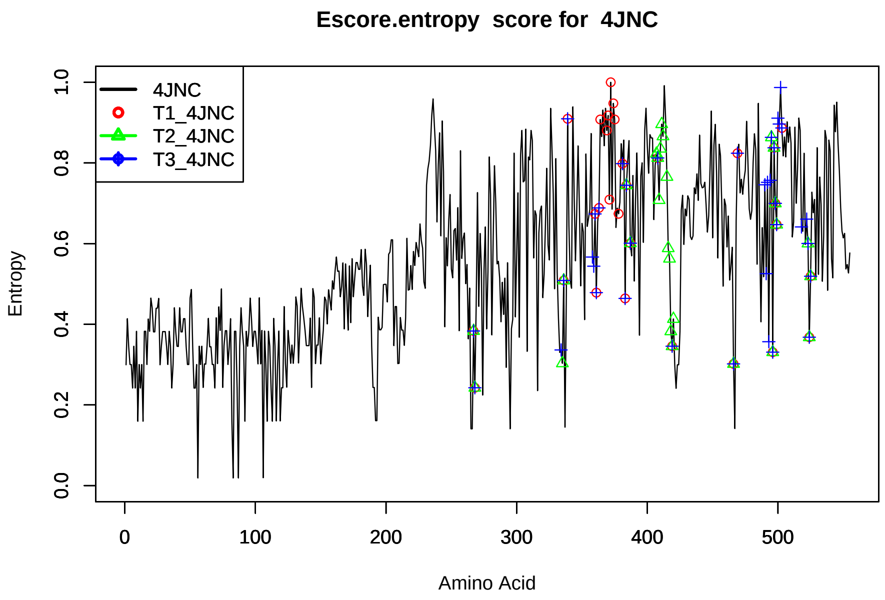

Figure 5: Plot shows the entropy (conservation) of the protein with the amino acids building structure marked on it. It facilitates the visual inspection of the chosen structural conservation in comparison with entire protein.

Compare the entropy metrics' performance and relationships on this particular dataset.

```
compare_cons_metrics(
  protein_entropy = prot_cons,
  structure_profile = profiles_for_structure,
  pdb_name = pdb_name
  )
```

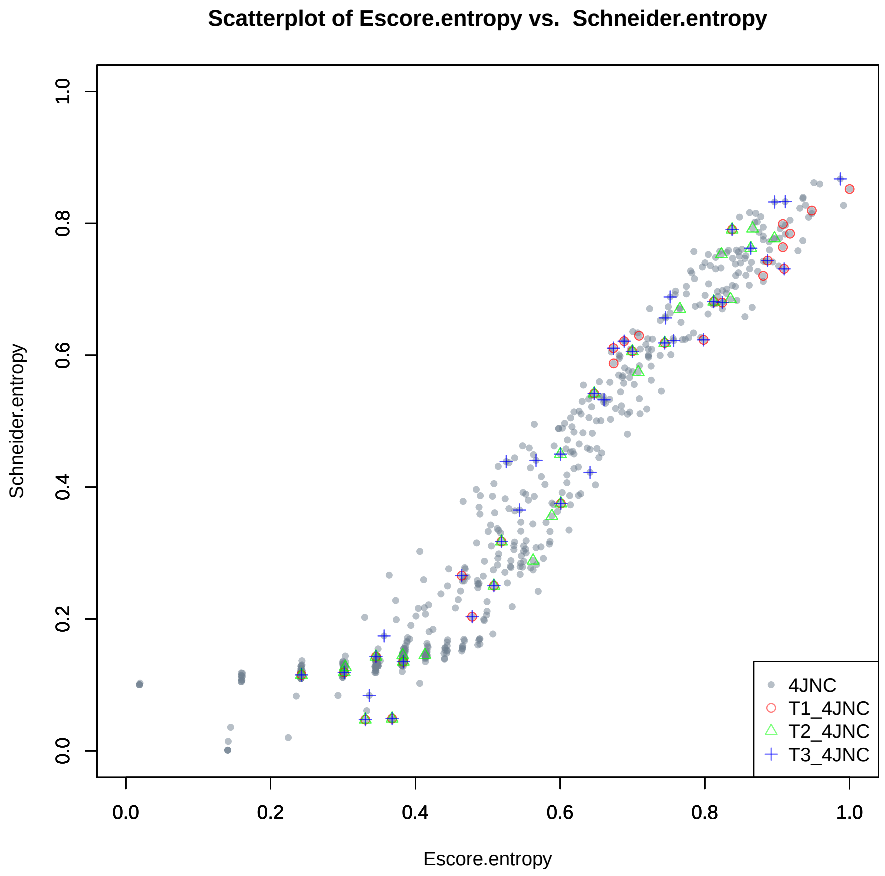

Figure 6: Scatterplot shows the relationship between a specified pair of entropy metrics.

Interpretation of results: the scatter plots facilitate the comparison and selection of the best metrics of conservation/entropy. In the exemplary case, the two metrics show that entropy for E_{score} metric allows for the distinction between values in the low entropy scope, which for Schneider score is rather indistinguishable. Simultaneously, E_{score} metric stays correlated with it in the rest of conservation score scope.

Perform the Kolmogorov-Smirnov non-parametric test to compare the samples: protein (without structure of interest) and the structure itself.

```
EQUAL = kolmogorov_smirnov_test(
  protein_entropy = list(Escore.entropy=prot_cons$Escore.entropy),
  structure_entropy = profiles_for_structure,
  alternative = 1,
  range = c(1:233),
  make_plot = T
  )
```

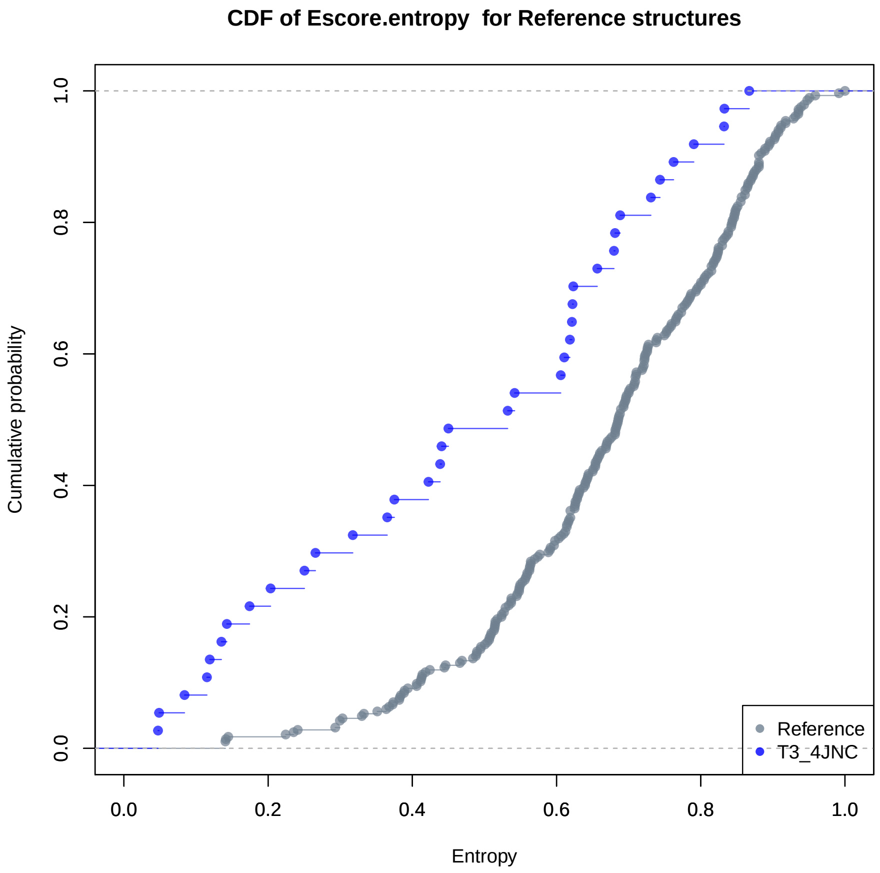

Figure 7: CDF of Escore entropy for reference structure.

Interpretation of results: The tunnel's CDF (cumulative distribution function) values are not different than values for the same entropy scores in the reference part of proteins (p=0.00041).

7 Grouping residues

Table 5: Summary of grouping used in BALCONY.

class	Substitution matrix	class	Size	class	Polarity and charges	class	Aromaticity
G1	A P S T	G1	A G S	G1	W S T Y N K	G1	F W H Y
G2	R Q E K	G2	V C D N T P	G1A	H R K	G2	others
G3	N D G H	G3	others	G1B	E D		
G4	I L M V			G2	others		
G5	F V Y						
G6	C						

Size according to [Valdar, 2002]

- *G1*: tiny
- *G2*: small
- *G3*: others

Side chain polarity and charges according to [Valdar, 2002]

- *G1*: polar
- *G1A*: polar and charged positively
- *G1B*: polar and charged negatively
- *G2*: others

Aromaticity according to [Valdar, 2002]

- *G1*: residues with aromatic ring
- *G2*: others

Substitution matrix: A hierarchical clustering of distances among amino acids in the BLOSUM62 substitution matrix [Henikoff and Henikoff, 1992] was performed. This method allowed the determination of evolutionary preferences for residue substitutions, which combines different residues properties. The effective clusters are presented on the dendrogram below:

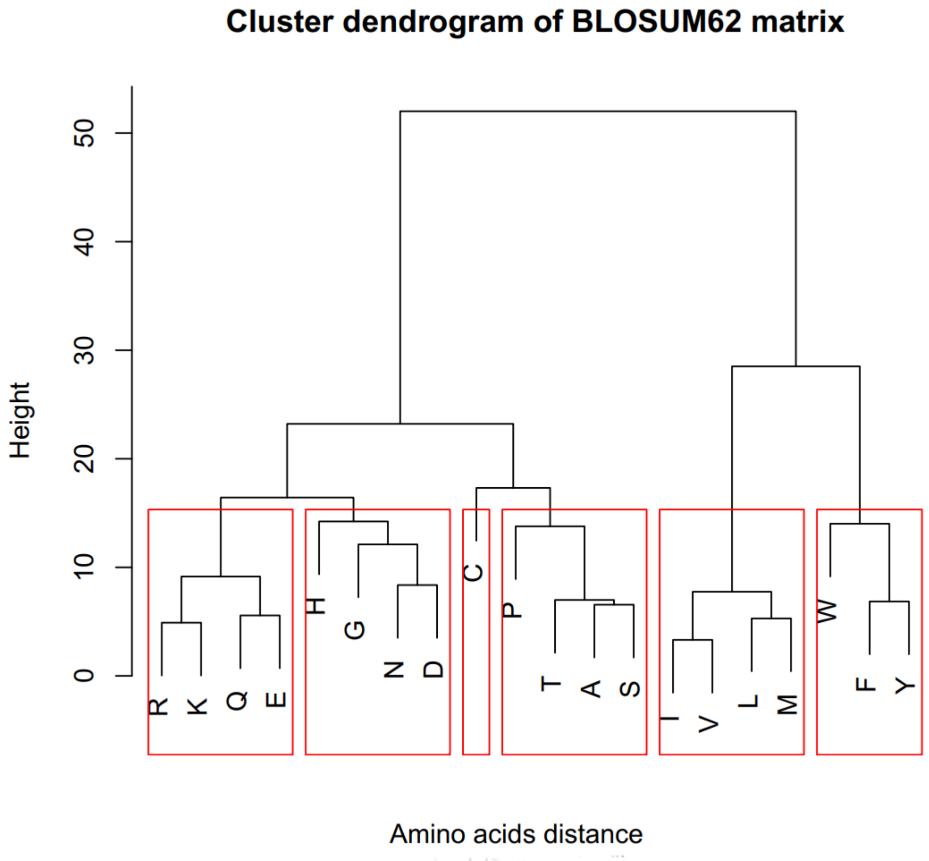

Figure 8: The dendrogram obtained after hierarchical clustering of the BLOSUM62 matrix for 20 amino acids available in the R package Biostrings, k=6, method: Ward minimum variance.

References

Atomic coordinate entry format version 3.3. URL http://www.wwpdb.org/documentation/file-format-content/format33/v3.3.html.

UniProt: the universal protein knowledgebase. *Nucleic Acids Research*, 45(D1):D158–D169, January 2017. ISSN 0305-1048. doi: 10.1093/nar/gkw1099. URL https://academic.oup.com/nar/article/45/D1/D158/2605721/UniProt-the-universal-protein-knowledgebase.

Helen Berman, Kim Henrick, and Haruki Nakamura. Announcing the worldwide Protein Data Bank. *Nature Structural & Molecular Biology*, 10(12):980–980, dec 2003. ISSN 1545-9993. doi: 10.1038/nsb1203-980. URL http://www.nature.com/articles/nsb1203-980.

Delphine Charif and Jean R. Lobry. SeqinR 1.0-2: A Contributed Package to the R Project for Statistical Computing Devoted to Biological Sequences Retrieval and Analysis. In Dr Ugo Bastolla, Professor Dr Markus Porto, Dr H. Eduardo Roman, and Dr Michele Vendruscolo, editors, *Structural Approaches to Sequence Evolution*, Biological and Medical Physics, Biomedical Engineering, pages 207–232. Springer Berlin Heidelberg, 2007.

Eva Chovancova, Antonin Pavelka, Petr Benes, Ondrej Strnad, Jan Brezovsky, Barbora Kozlikova, Artur Gora, Vilem Sustr, Martin Klvana, Petr Medek, Lada Biedermannova, Jiri Sochor, and Jiri Damborsky. CAVER 3.0: A Tool for the Analysis of Transport Pathways in Dynamic Protein Structures. *PLOS Computational Biology*, 8(10):e1002708, October 2012. ISSN 1553-7358. doi: 10.1371/journal.pcbi.1002708. URL http://journals.plos.org/ploscompbiol/article?id=10.1371/journal.pcbi.1002708.

Jean Michel Claverie. Some useful statistical properties of position-weight matrices. *Computers and Chemistry*, 18(3):287–294, sep 1994. ISSN 00978485. doi: 10.1016/0097-8485(94)85024-0. URL https://www.sciencedirect.com/science/article/pii/0097848594850240http://linkinghub.elsevier.com/retrieve/pii/0097848594850240.

S. R. Eddy. Profile hidden Markov models. *Bioinformatics (Oxford, England)*, 14(9):755–763, 1998. ISSN 1367-4803.

S. S. Hannenhalli and R. B. Russell. Analysis and prediction of functional sub-types from protein sequence alignments. *Journal of Molecular Biology*, 303(1):61–76, October 2000. ISSN 0022-2836. doi: 10.1006/jmbi.2000.4036.

Jorja G. Henikoff and Steven Henikoff. Using substitution probabilities to improve position-specific scoring matrices. *Bioinformatics (Computer applications in the biosciences: CABIOS)*, 12(2):135–143, apr 1996. ISSN 1367-4803 (0266-7061). doi: 10.1093/bioinformatics/12.2.135. URL https://pdfs.semanticscholar.org/4c7d/e7ec837ba829640ae59121e6a815748f5c40.pdfhttps://academic.oup.com/bioinformatics/article-lookup/doi/10.1093/bioinformatics/12.2.135.

S Henikoff and J G Henikoff. Amino acid substitution matrices from protein blocks. *Proceedings of the National Academy of Sciences of the United States of America*, 89(22):10915–10919, November 1992. ISSN 0027-8424. URL http://www.ncbi.nlm.nih.gov/pmc/articles/PMC50453/.

S Henikoff and Jorja G. Henikoff. Position-based sequence weights. *Journal of molecular biology*, 243(4):574–8, nov 1994. ISSN 0022-2836. URL http://www.ncbi.nlm.nih.gov/pubmed/7966282.

Ralf Landgraf, Daniel Fischer, and David Eisenberg. Analysis of heregulin symmetry by weighted evolutionary tracing. *Protein Engineering, Design and Selection*, 12(11):943–951, November 1999. ISSN 1741-0126. doi: 10.1093/protein/12.11.943. URL https://academic.oup.com/peds/article/12/11/943/1489691/Analysis-of-heregulin-symmetry-by-weighted.

I. Mihalek, I. Reš, and O. Lichtarge. A family of evolution–entropy hybrid methods for ranking protein residues by importance. *Journal of Molecular Biology*, 336(5):1265 – 1282, 2004. ISSN 0022-2836. doi: https://doi.org/10.1016/j.jmb.2003.12.078. URL http://www.sciencedirect.com/science/article/pii/S0022283604000245.

H. Pagès, P. Aboyoun, R. Gentleman, and S. DebRoy. *Biostrings: String objects representing biological sequences, and matching algorithms*, 2016. R package version 2.42.1.

Chris Sander and Reinhard Schneider. Database of homology-derived protein structures and the structural meaning of sequence alignment. *Proteins: Structure, Function, and Bioinformatics*, 9(1):56–68, January 1991. ISSN 1097-0134. doi: 10.1002/prot.340090107. URL http://onlinelibrary.wiley.com/doi/10.1002/prot.340090107/abstract.

C. E. Shannon. A Mathematical Theory of Communication. *Bell System Technical Journal*, 27(3):379–423, July 1948. ISSN 1538-7305. doi: 10.1002/j.1538-7305.1948.tb01338.x. URL http://onlinelibrary.wiley.com/doi/10.1002/j.1538-7305.1948.tb01338.x/abstract.

Reema K. Thalji, Jeff J. McAtee, Svetlana Belyanskaya, Martin Brandt, Gregory D. Brown, Melissa H. Costell, Yun Ding, Jason W. Dodson, Steve H. Eisennagel, Rusty E. Fries, Jeffrey W. Gross, Mark R. Harpel, Dennis A. Holt, David I. Israel, Larry J. Jolivette, Daniel Krosky, Hu Li, Quinn Lu, Tracy Mandichak, Theresa Roethke, Christine G. Schnackenberg, Benjamin Schwartz, Lisa M. Shewchuk, Wensheng Xie, David J. Behm, Stephen A. Douglas, Ami L. Shaw, and Joseph P. Marino Jr. Discovery of 1-(1,3,5-triazin-2-yl)piperidine-4-carboxamides as inhibitors of soluble epoxide hydrolase. *Bioorganic & Medicinal Chemistry Letters*, 23(12):3584–3588, June 2013. ISSN 0960-894X. doi: 10.1016/j.bmcl.2013.04.019. URL http://www.sciencedirect.com/science/article/pii/S0960894X13004885.

William S. J. Valdar. Scoring residue conservation. *Proteins*, 48(2):227–241, August 2002. ISSN 1097-0134. doi: 10.1002/prot.10146.

Tai Te Wu and Elvin A. Kabat. An Analysis of the Sequences of the Variable Regions of Bence Jones Proteins and Myeloma Light Chains and Their Implications for Antibody Complementarity. *Journal of Experimental Medicine*, 132(2):211–250, August 1970. ISSN 0022-1007, 1540-9538. doi: 10.1084/jem.132.2.211. URL http://jem.rupress.org/content/132/2/211.
